# Evaluation of Biological Response of STRO-1/c-Kit Enriched Human Dental Pulp Stem Cells to Titanium Surfaces Treated with Two Different Cleaning Systems

**DOI:** 10.3390/ijms20081868

**Published:** 2019-04-16

**Authors:** Enrico Conserva, Alessandra Pisciotta, Laura Bertoni, Giulia Bertani, Aida Meto, Bruna Colombari, Elisabetta Blasi, Pierantonio Bellini, Anto de Pol, Ugo Consolo, Gianluca Carnevale

**Affiliations:** 1 Department of Surgery, Medicine, Dentistry and Morphological Sciences with interest in Transplant, Oncology and Regenerative Medicine, University of Modena and Reggio Emilia, 41125 Modena, Italy; enrico.conserva@unimore.it (E.C.); alessandra.pisciotta@unimore.it (A.P.); laura.bertoni@unimore.it (L.B.); giulia.bertani@unimore.it (G.B.); aida.meto@unimore.it (A.M.); bruna.colombari@unimore.it (B.C.); elisabetta.blasi@unimore.it (E.B.); pierantonio.bellini@unimore.it (P.B.); anto.depol@unimore.it (A.d.P.); ugo.consolo@unimore.it (U.C.); 2 Operative Unit of Dentistry and Maxillofacial Surgery, Department Integrated Activity-Specialist Surgeries, University-Hospital of Modena, 41125 Modena, Italy

**Keywords:** human dental pulp stem cells, stemness properties, titanium surface properties

## Abstract

Peri-implantitis—an infection caused by bacterial deposition of biofilm—is a common complication in dentistry which may lead to implant loss. Several decontamination procedures have been investigated to identify the optimal approach being capable to remove the bacterial biofilm without modifying the implant surface properties. Our study evaluated whether two different systems—Ni-Ti Brushes (Brush) and Air-Polishing with 40 µm bicarbonate powder (Bic40)—might alter the physical/chemical features of two different titanium surfaces—machined (MCH) and Ca^++^ nanostructured (NCA)—and whether these decontamination systems may affect the biological properties of human STRO-1^+^/c-Kit^+^ dental pulp stem cells (hDPSCs) as well as the bacterial ability to produce biofilm. Cell morphology, proliferation and stemness markers were analysed in hDPSCs grown on both surfaces, before and after the decontamination treatments. Our findings highlighted that Bic40 treatment either maintained the surface characteristics of both implants and allowed hDPSCs to proliferate and preserve their stemness properties. Moreover, Bic40 treatment proved effective in removing bacterial biofilm from both titanium surfaces and consistently limited the biofilm re-growth. In conclusion, our data suggest that Bic40 treatment may operatively clean smooth and rough surfaces without altering their properties and, consequently, offer favourable conditions for reparative cells to hold their biological properties.

## 1. Introduction

The use of dental implants in daily clinical practice is currently widespread and in huge growth: the modern implant therapy allows in fact not only to offer a biological and functional advantage for many patients, compared with fixed or removable prosthetic solutions but also to obtain excellent long term results, as confirmed by previous studies reporting survival rates of 95.7% and 92.8% after 5 and 10 years, respectively [1]. However, despite these high survival rates, implant rehabilitation can fail. The most relevant troubles concerning osseointegrated implants are the peri-implant diseases, such as mucositis (reversible) and peri-implantitis (not reversible). Peri-implantitis has been defined as a disease with infectious pathogenesis characterized by a mucosal lesion often associated with bleeding, suppuration, increased probing depth always accompanied by marginal bone loss [2]. In this context, several factors affecting the systemic health status of the patients, that is, chronic diseases, autoimmune/inflammatory diseases, in combination with poor oral hygiene and smoking might influence the host-microbial interface very early in the healing phase following dental implant thus increasing the risk of peri-implant diseases [3].

The formation and organization of a bacterial biofilm on the implant portions exposed to the oral cavity is the initial cause of the peri-implant diseases and its removal is the goal to prevent or treat these diseases. Microbial biofilm commonly occurs in the oral cavity; it consists of a multispecies community, including Gram+ and Gram− bacteria as well as fungal cells, organized as sessile cells, tightly embedded in a matrix of polysaccharide origin. Biofilm formation and development is a process that begins with adhesion of initially planktonic microorganisms, followed by growth, extracellular polymeric matrix production, detachment and delocalization. Microbial biofilm occurs both in biotic and abiotic surfaces; once structured, biofilm-associated cells acquire enhanced resistance to cleaning treatment, antimicrobial drugs and host immune defences with respect to their planktonic counterparts [4]. To date, several methods have been developed with the aim of decontaminating implants: mechanical systems (Titanium brushes, Air-polishing systems, Ultra-Subsonic systems, Laser, Curettes) and chemical systems or antimicrobial solutions (Chlorhexidine in solution or in gel, Stannous fluoride, Tetracycline, Minocycline, Citric acid, Hydrogen peroxide and Gel etching with 35% phosphoric acid) [5,6,7]. Most of these decontaminating treatments might alter the chemical-physical properties of the implant surface [8,9,10,11]. Therefore, the ideal decontamination system is expected to be capable of breaking and removing the bacterial biofilm, without modifying the surface properties and thus maintaining the implant surface biologically favourable to adhesion and differentiation of the reparative cells grown onto the implant. It is well known indeed that the micro/nanotopography and the chemical composition of implant surfaces might influence the osseointegration process, by affecting and modifying the biological properties of the cells that interact with the surface [12,13,14,15]. Based on these considerations, among several decontamination techniques investigated, Nickel Titanium (Ni-Ti) brushes and Air Polishing systems have aroused interest. Ni-Ti is a resistant material with high flexibility when subjected to heating/cooling alternations. Moreover, the centrifugal force of the motor movement, the pressure exerted by the operator’s hand and the heating/cooling alternations allow the brush to effectively reach even the most difficult spaces to clean. As a matter of fact, Ni-Ti brushes proved more effective in removing plaque than steel or plastic curettes, being, at the same time, less aggressive towards the implant surfaces [16,17,18,19]. Likewise, air-polishing devices based on the use of bicarbonate powder, which historically represents the first type of substance used in this approach, were shown to be highly effective in the mechanical removal of biofilm from different surface types, such as machined, SLA, sandblasted and TPS, with previous data showing that this approach provided better outcomes when compared to other instruments, such as plastic inserts of sonic devices [9,20,21].

Human DPSCs can be easily obtained from routine tooth extraction procedures and own self-renewal properties and a high regenerative potential [22]. Although dental pulp stem cells are a heterogeneous cell population, the immune selection against the stemness markers c-Kit and STRO-1 allows to isolate an adequate source of pre-osteoblast/odontoblast cells [23,24] which represent the ideal cell candidate in order to mimic in vitro the physiological processes of osseointegration and to investigate how different cleansing approaches might modify the cells-implants interactions.

The aim of this study was to investigate whether the use of two different mechanical decontamination systems, namely Ni-Ti Brushes and Air-Polishing System with 40 µm bicarbonate powder, might alter the roughness and chemical composition of two different implant surfaces (Machined and Ca^++^ Nano-incorporated) and whether the two decontamination systems may impact the biological and stemness features of DPSCs as well as the bacterial ability to produce biofilm.

## 2. Results

### 2.1. Titanium Surface Characterization

**Figure 1:**
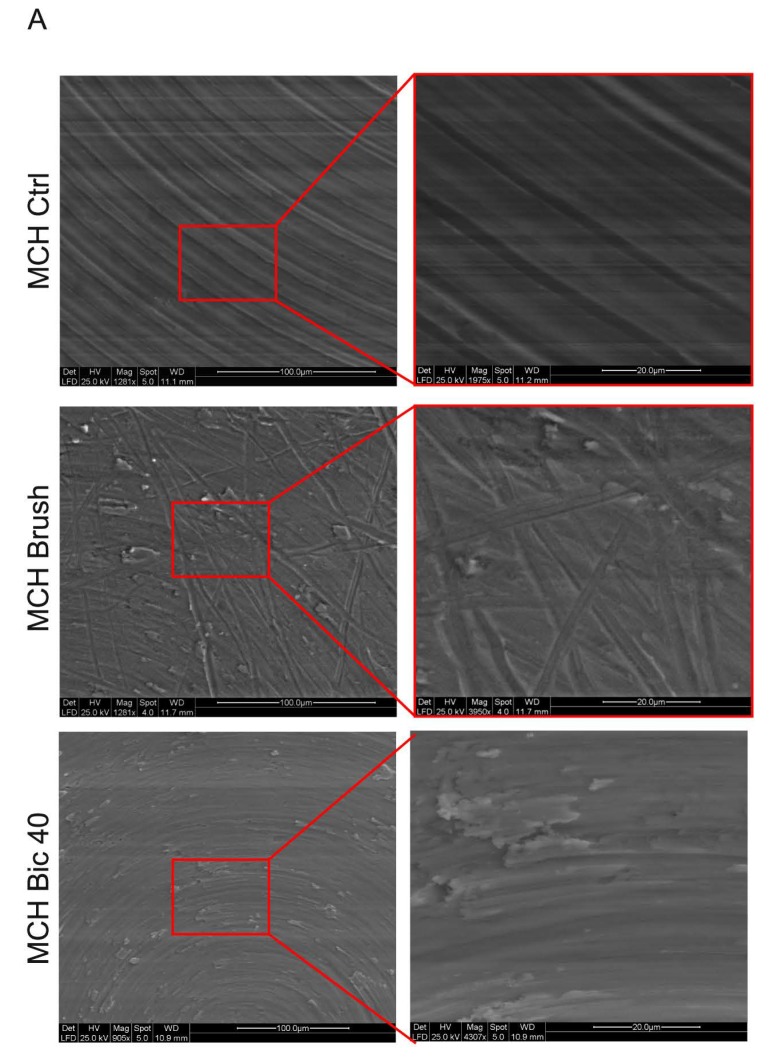

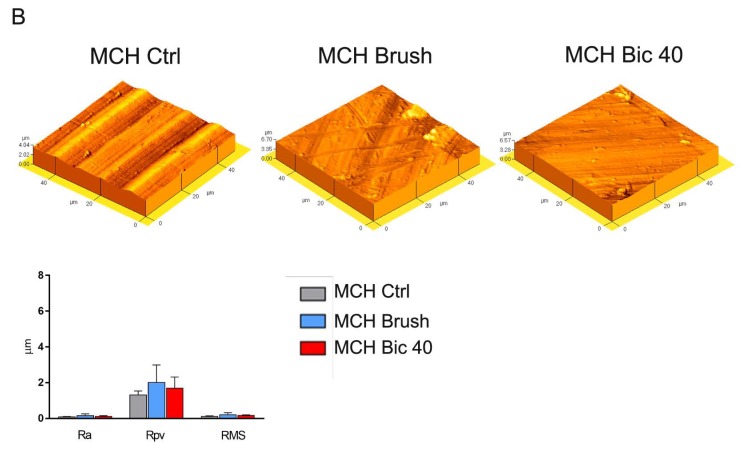
Machined (MCH) surfaces characterization after cleaning treatments. (**A**) Scanning electron microscopy (SEM) analysis at different magnifications was carried out on MCH titanium surfaces from the three experimental groups (Ctrl, Brush and Bic40, as indicated) in order to evaluate the surface topography. Scale bars: 100 µm (left) and 20 µm (magnifications on the right). (**B**) Atomic force microscopy (AFM) analysis of MCH surfaces. Histograms report the surface roughness expressed as Ra, Rpv and Rsm values. Values represent mean ± SD of three independent experiments; one-way ANOVA followed by Newman-Keuls post-hoc test.

Scanning electron microscopy (SEM) analysis carried out on MCH surfaces from each experimental group is shown in Figure 1A. At lower magnifications, MCH control surface displayed concentric irregularities according to our previous findings [15]. The polishing treatment with Ni-Ti brush was characterized by grooves oriented in all the directions through the entire surface of the disks, as reported in either lower and higher magnifications. On the MCH titanium surfaces treated with Bic40, the presence of slight alterations of the whole surface was observed. Particularly, newly formed irregularities were detected on the entire treated disk (Figure 1A).

**Figure 2:**
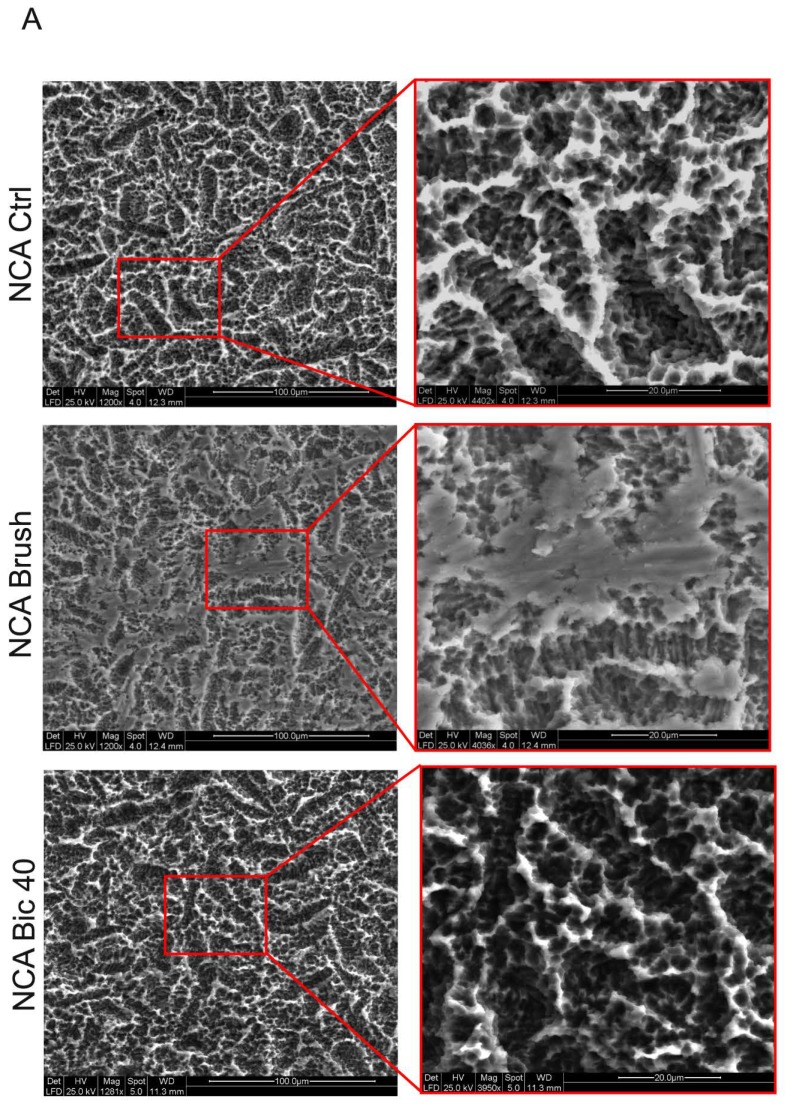

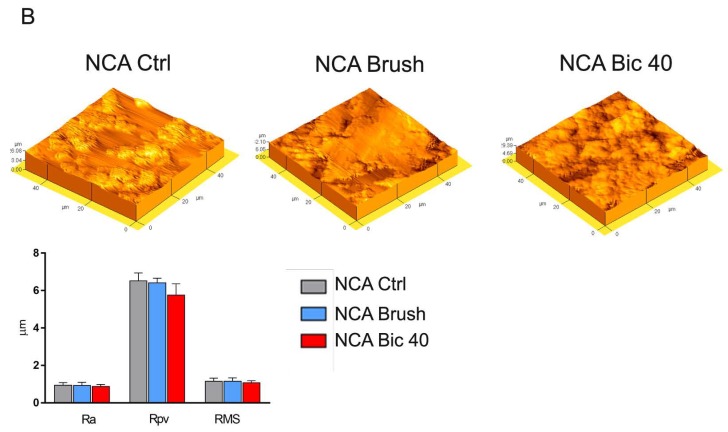
NCA surfaces characterization after cleansing treatments. (**A**) SEM analysis at different magnifications was carried out on NCA titanium surfaces from the three experimental groups (Ctrl, Brush and Bic40, as indicated) in order to evaluate the surface topography. Scale bars: 100 µm (left) and 20 µm (magnifications on the right). (**B**) AFM analysis of NCA surfaces. Histograms report the surface roughness expressed as Ra, Rpv and Rsm values. Values represent mean ± SD of three independent experiments. No statistically significant difference was detected among the groups; one-way ANOVA followed by the Newman-Keuls post-hoc test.

Furthermore, atomic force microscopy (AFM) analysis was performed to evaluate the roughness of MCH disks following the different polishing treatments. In particular, as shown in Figure 1B, Ra, Rpv and Rsm were determined for each experimental group. With regard to Rpv parameter, higher values were recorded in MCH Brush group, in comparison to control MCH and MCH Bic40, although these differences were not statistically significant (Figure 1B). At the same time, SEM analysis was carried out on NCA surfaces from the three experimental groups. Data are reported in Figure 2A. According to previous data from our group [15], control NCA were characterized by homogeneous irregularities spread through the whole analysed area. The polishing treatment with Brush induced a notable modification of the surface: particularly, a flattening of the peaks typical of NCA surface was observed at lower and higher magnifications. Conversely, the air-polishing treatment with Bic40 did not induce any relevant alteration of the nanotopography of the surface. These observational data were not confirmed by AFM analyses, as a matter of fact, Ra, Rpv and Rms parameters did not differ among the three experimental groups. Likely, this evidence might be due to the AFM instrumental sensitivity (Figure 2B). Taken together, data on surface roughness of MCH and NCA surfaces treated with Bic40 did not show any significant difference, in terms of nanotopography, from both MCH and NCA controls.

### 2.2. Stem Cells Morphology and Proliferation on Titanium Surfaces after Polishing Treatments

**Figure 3:**
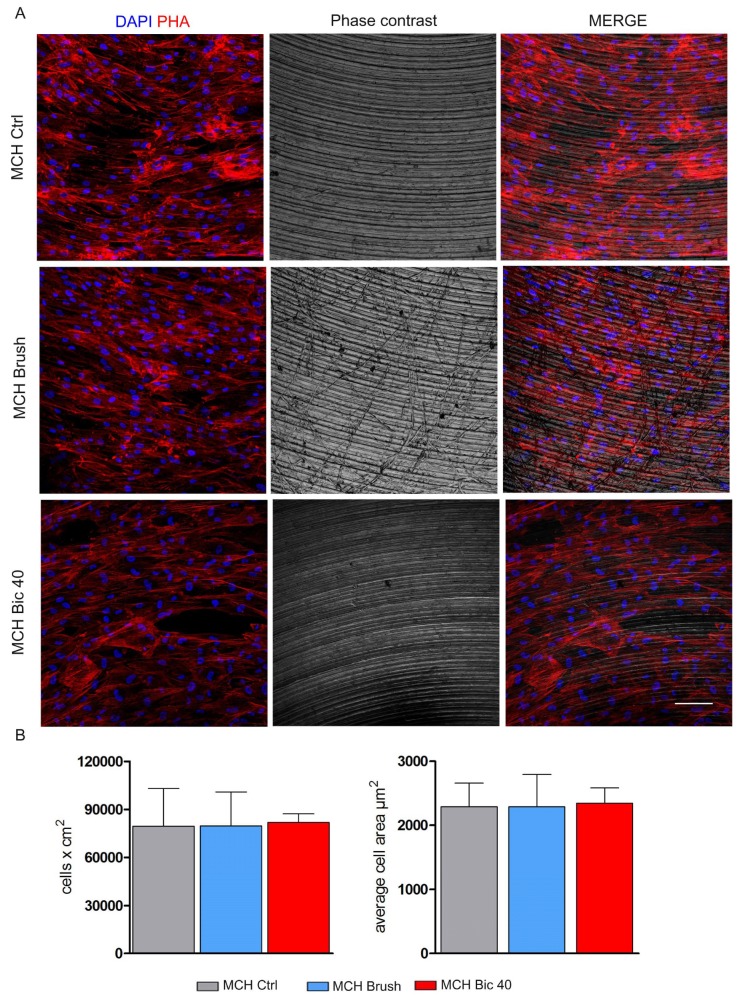
Evaluation of hDPSCs morphology and proliferation on MCH titanium surfaces. (**A**) hDPSCs morphology was assessed by confocal microscopy with phalloidin staining after 7 days of culture on MCH Ctrl, MCH Brush and MCH Bic40 surfaces. (**B**) Cell proliferation on titanium disks was measured by counting cell nuclei after DAPI staining. Histograms show cell numbers after 7 days of culture on titanium surfaces from the three experimental groups. Values represent mean ± SD. No statistically significant difference was detected among the groups; one-way ANOVA followed by the Newman-Keuls post-hoc test. Scale bar: 100 μm.

**Figure 4:**
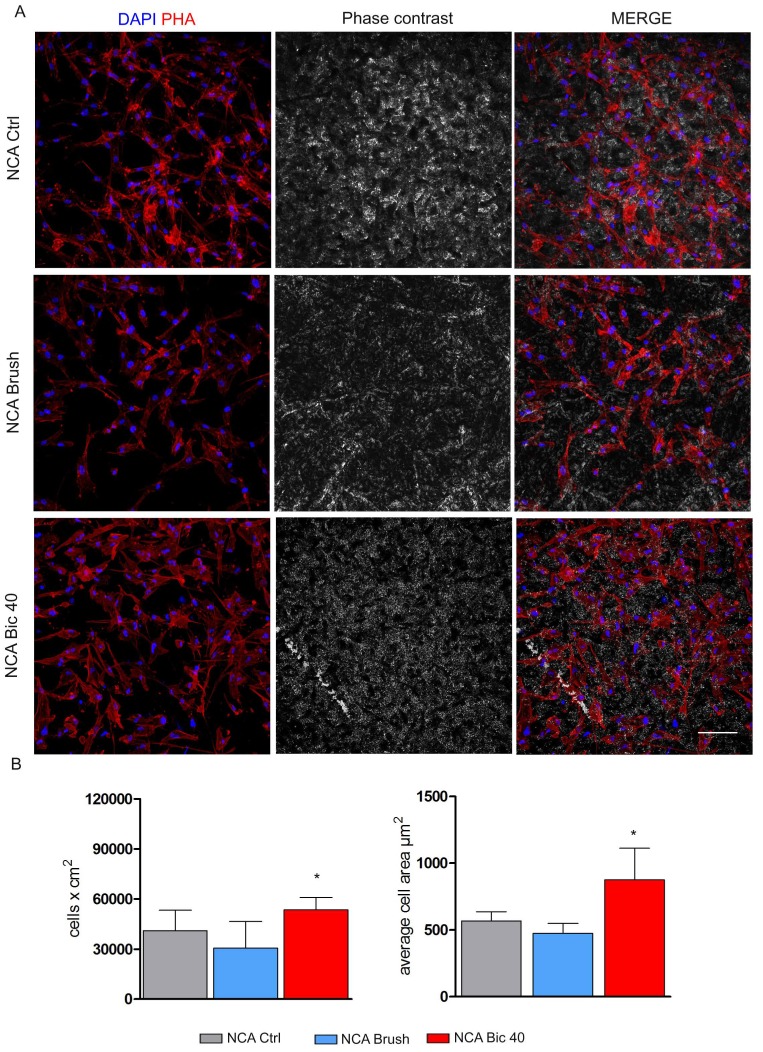
Evaluation of hDPSCs morphology and proliferation on NCA titanium surfaces. (**A**) hDPSCs morphology was assessed by confocal microscopy with phalloidin staining after 7 days of culture on NCA Ctrl, NCA Brush and NCA Bic40 surfaces. (**B**) Cell proliferation on titanium disks was measured by counting cell nuclei after DAPI staining. Histograms show cell numbers and average cell area after 7 days of culture on titanium surfaces from the three experimental groups. Values represent mean ± SD. * *p* < 0.05 NCA Bic 40 vs. NCA Brush, NCA Bic40 vs. NCA Ctrl; one-way ANOVA followed by the Newman-Keuls post-hoc test. Scale bar: 100 μm.

The hDPSCs morphology was evaluated by confocal microscopy, as shown in Figure 3 and Figure 4. Cells were stained with phalloidin and DAPI. After 7 days of culture under standard conditions on MCH surfaces, hDPSCs displayed a fibroblast-like morphology with cells being arranged parallel to the surface grooves without showing significant differences following the Brush and Bic40 polishing treatments. With regard to the distribution of cells through the surface area, we noticed that hDPSCs cultured on MCH Brush oriented not only along the grooves due to the industrial fabrication but also along the scratches created after the Brush cleaning. No differences were observed in hDPSCs seeded on MCH Bic40 when compared to the control group (Figure 3A). Also, no differences in terms of proliferation rate were observed among the three experimental groups as indicated by histograms (Figure 3B).

Figure 4 shows the morphology, distribution and proliferation of hDPSCs after 7 days of culture on NCA surfaces following Brush and Bic40 treatments. As formerly described, the culture on NCA disks induced a morphology alteration in hDPSCs. In particular, cells were homogeneously spread through the whole area and showed an irregular shape with reduction of the average cell area. The same features were observed in hDPSCs cultured on NCA Brush disks. Interestingly, when NCA disks were cleaned with Bic40 hDPSCs grew still homogeneously although recovering their typical fibroblast-like morphology, as reported when cultured on MCH disks. This shift in morphology was reflected also by an increased proliferation rate and by values of average cell area of hDPSCs cultured on NCA Bic40, with respect to NCA Ctrl and NCA Brush (* *p* < 0.05, Figure 4B).

### 2.3. Expression of Stemness Markers

Human DPSCs were immune-selected against STRO-1 and c-Kit. After 7 days of culture on MCH and NCA surfaces, immunofluorescence analysis was performed in order to evaluate the maintenance of their biological properties. The expression of STRO-1 and c-Kit, two typical mesenchymal stem cells markers, were investigated in MCH and NCA after cleaning treatments.

**Figure 5:**
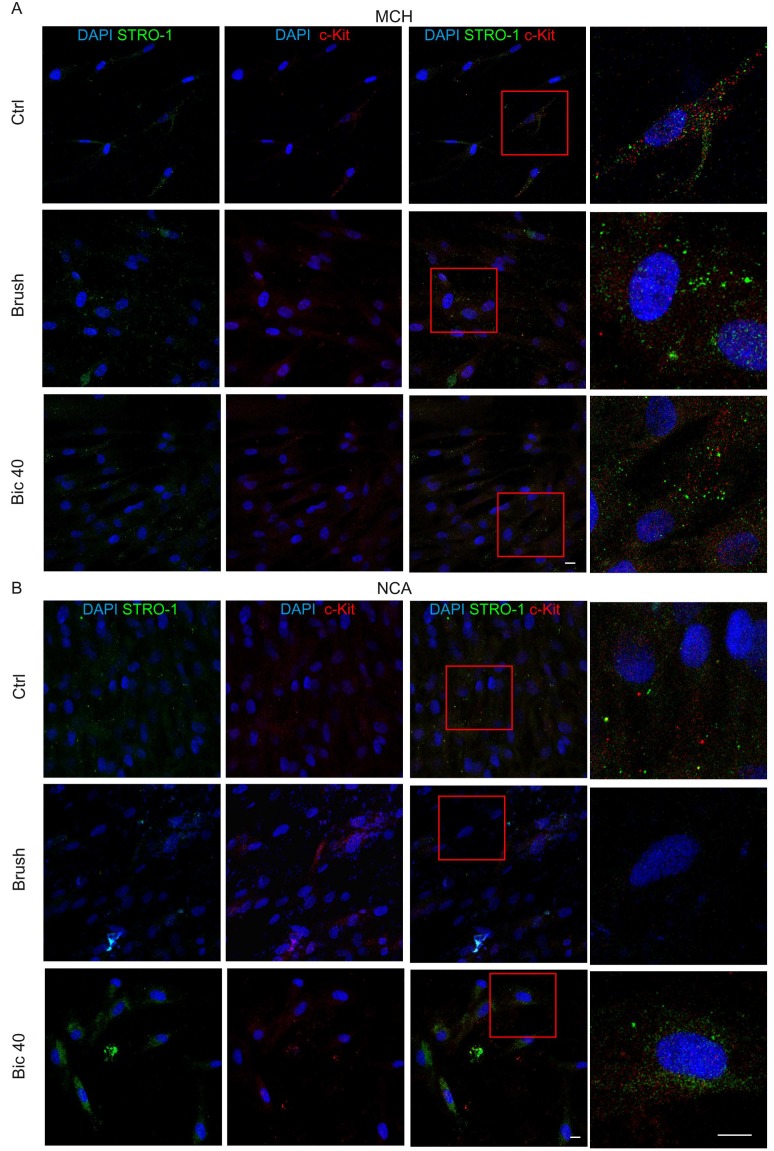
Evaluation of stemness markers of hDPSCs on titanium surfaces after cleaning treatments. Immunofluorescence analysis of STRO-1 and c-Kit in hDPSCs cultured for 7 days on MCH (**A**) and NCA (**B**) surfaces from the three experimental groups. Red squares indicate details reported at higher magnifications on the right. Nuclei were stained with DAPI. Scale bar: 10 μm.

As reported in Figure 5, hDPSCs cultured on MCH Ctrl showed the expression of both mesenchymal markers. These markers were still observed in hDPSCs cultured either on MCH Brush and MCH Bic40.

On the contrary, we noticed a reduction in STRO-1 and c-Kit expression in hDPSCs grown on NCA Ctrl and NCA Brush after 7 days of culture. Conversely, the expression of these markers was evident in hDPSCs cultured on NCA Bic40. The morphology data in association with stemness evaluation indicate that after cleansing with Bic40, the NCA surface appeared more favourable/suitable to the growth of hDPSCs in their physiological microenvironment.

### 2.4. Microbial Biofilm Formation onto Titanium Disks

**Figure 6:**
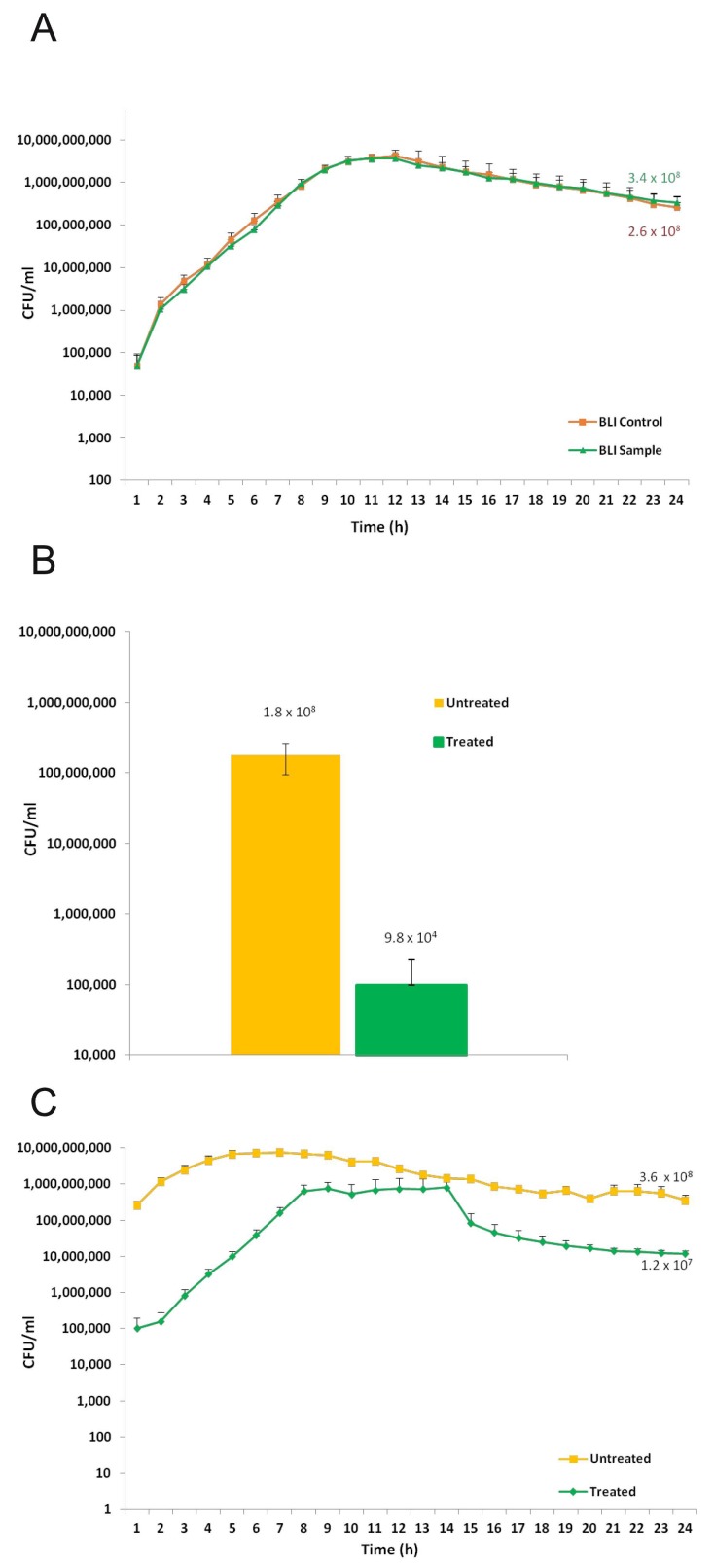
*P. aeruginosa* growth and biofilm formation on titanium disks, treated or not with Bic40 procedure, as assessed by a bioluminescent (BLI) bacterial strain. (**A**) *P. aeruginosa* growth was not affected by the presence of titanium disks. BLI-*Pseudomonas* (10^6^/mL) in TSB plus 2% sucrose was seeded in 96 black well-plate and incubated at 35 °C in the presence (orange) or absence (green) of titanium disks (1/well). The plates were then incubated by Fluoroskan and the bioluminescent signal was recorded in real time (h), up to 24 h. By the calibration curve, the RLUs were converted in CFU/mL + SEM (Standard Error Mean), as indicated in the Y axis. (**B**) Effects of the cleaning procedure on titanium disks-associated biofilm. Microbial biofilm produced onto titanium disks (by 24 h incubation, as above) was exposed or not to the Bic40 cleaning procedure (treated vs. untreated group); the residual bioluminescent signal was measured and converted, by the calibration curve, in CFU/mL ± SEM, as indicated in the Y axis. (**C**) Real time monitoring of *P. aeruginosa* re-growth on treated and untreated titanium disks. The microbial re-growth, occurring in Bic40-treated and untreated disks, was evaluated in real time, for additional 24 h. Then, the bioluminescent signal was measured and converted in CFU, returning values of 3.6 × 10^8^/mL and 1.2 × 10^7^/mL, in untreated and treated groups, respectively.

Based on the biological results concerning morphology, proliferation and stemness markers, it was then evaluated whether the polishing treatment with Bic40 on NCA surface may affect the microbial growth. Thus, *Pseudomonas aeruginosa* (10^6^/mL) was seeded at time 0, in two sets of wells, containing or not the titanium disks; then, the plate was incubated at 35 °C for 24 h and microbial growth was assessed by bioluminescent analysis. As shown in Figure 6A, a superimposable trend was observed in the two groups of wells (control: no disks; sample: with disks); moreover, at 24 h, comparably high levels of total microbial load were achieved, being 2.6 × 10^8^/mL and 3.4 × 10^8^/mL the CFU/mL detected in control and sample groups, respectively. These data indicated that the presence of titanium disks did not affect microbial growth. To assess the occurrence of biofilm onto such disks, the latter were exposed to bacteria for 24 h, washed twice with PBS to eliminate the non-adherent microbial cells and then the residual bioluminescent signal was evaluated, as measure of formed biofilm. The obtained bioluminescent signal was converted in CFU and indicated that biofilm was produced at amounts as high as 1.95 × 10^8^ CFU/mL (data not shown).

### 2.5. Microbial Biofilm Removal from Titanium Disks

To assess the efficacy of the Bic40 cleaning procedure against a preformed biofilm, 4 disks containing a 24 h-old biofilm were treated with Bic40 procedure, as detailed above (treated group), while 3 disks, used as controls were not treated (untreated group); then, each disk was transferred in a new well with fresh medium and incubated for additional 24 h. As shown in Figure 6B, when compared to the untreated groups, the Bic40-treated disks displayed an about 3 logs drop in terms of CFU/mL.

### 2.6. Microbial Re-Growth on Treated and Untreated Titanium Disks

As detailed in Figure 6C, the microbial load in the untreated group remained almost constant during the subsequent 24 h range. In contrast, treated disks showed a delayed and time-related increase in CFU. In particular, the major differences between treated and untreated disks occurred within the first 5–6 h; then, microbial load on treated disks reached a plateau level, that consistently remained about 1 log below the values observed in the untreated disks until at 24 h.

## 3. Discussion

The peri-implant disease is due to the colonization of the implant surfaces by pathogens which constitute a biofilm [25]. Bacterial adhesion and biofilm formation play a fundamental role not only into the pathogenesis of peri-implantitis but also into the implant survival [12,13,26]. In a previous study [27] we observed that the biofilm occurred regardless of the degree of surface roughness. Therefore, the removal of the biofilm from the implant surface, indistinctly smooth or rough, is the primary objective. It is well known that the response of cells and tissues to a biomaterial depends on the properties of the material itself such as surface topography, chemical composition and capability to interact with body fluids [15,28]. Following bacterial contamination, the procedures used to decontaminate the implant surface can cause alterations of its topography and its chemical composition. To this regard and to the best of our knowledge the optimal cleansing approach is expected to effectively remove the bacteria biofilm without altering the chemical and physical properties of the implant and consequently the biological properties of cells involved in the osseointegration process. In this study we analysed two different titanium surfaces, before and after the treatment with two cleansing approaches, mimicking what physiologically occurs in terms of cells/implant interactions after decontamination procedures.

Qualitative analysis of surface morphology revealed how in both MCH and NCA groups the surfaces treated with Ni-Ti brushes are morphologically different from the untreated surfaces, in agreement with Park et al. [18]. In fact, SEM analysis showed the presence of deep grooves heterogeneously distributed over the MCH surface and flattened area over the NCA surface. Slight although not statistically significant differences were revealed by Ra, Rpv and Rsm physical parameters. Conversely, the treatment with Air-polishing system with Bic40 produced not significant alterations of both the MCH and NCA surfaces in terms of physical parameters. Subsequently, the aim of the study was to evaluate the biological features of stem cells that embryologically participate to the osseointegration process. The use of dental pulp stem cells represents a suitable choice in terms of stemness properties and ease of isolation. Although hDPSCs are a heterogeneous population, we used a stem cell population enriched for the expression of the stemness markers STRO-1 and c-Kit, which represent a strictly mesenchymal origin able to differentiate in bone, adipose and myogenic lineages. To the best of our knowledge, we noticed that on MCH titanium surface before and after both the cleansing treatments, hDPSCs maintained their fibroblast-like morphology without any alteration of cell proliferation. The only difference observed concerned the hDPSCs distribution pattern through the MCH Brush surface, in fact cells were spread along the grooves produced by Ni-Ti brush. On the contrary, hDPSCs grown on MCH Bic40 and MCH ctrl surfaces did not show any difference.

Regarding NCA surface, a change in cell morphology was observed in NCA ctrl and NCA brush, in accordance with our previous findings [15]. After the treatment with Bic40 hDPSCs showed their typical cell morphology and also demonstrated a statistically significant higher proliferation rate, when compared to NCA ctrl and NCA Brush surfaces: this phenomenon might be attributed to the interaction of calcium ions incorporated on the NCA surface with bicarbonate ions from the cleansing treatment with Bic40. These observations were confirmed by the evaluation of stemness markers on MCH and NCA surfaces before and after the cleansing treatments. In particular, whereas no differences were detected in any MCH group, we noticed that STRO-1 and c-Kit expression were maintained in NCA Bic40 group. As a matter of fact, the maintenance of stemness is a primary requirement to preserve the biological properties including self-renewal and differentiation capabilities and immunomodulatory properties and consequently to avoid cell senescence. Based on these results, Bic40 might represent the most suitable cleansing treatment. To further confirm the efficacy of Bic40, we also performed microbiology assays. Using a recently established model precious in assessing microbial biofilm formation onto medical devices [29], here we showed that BLI-*Pseudomonas* has the ability to adhere to the titanium disks and to form a consistent biofilm on their surface. Moreover, the cleaning treatment with BIC 40 µm is capable of reducing the biofilm of about 99% with respect to untreated control group (100% vs. 0.05%, respectively). In particular, the microbial load, evaluated as RLU and converted in CFU/mL, has been reduced of more than 3 logs in the treated groups as compared to their controls. Furthermore, microbial re-growth in treated disks remains consistently below the control values (difference of about 1 log). We may hypothesize that the combination of the physical treatment (dry spray) and the hypertonic condition (sodium bicarbonate accumulation onto titanium disks) negatively impact on microbial cell-viability. Furthermore, the enrichment of air-polishing powders with antimicrobial fillers such as Ciprofloxacin and/or mucosal defensive agents such as Zinc L-carnosine might improve the antibacterial action of the cleaning tool and its biocompatibility towards soft tissues [30]. In conclusion, we demonstrated that Bic40 provides a suitable cleansing approach either on smooth and rough surfaces.

## 4. Materials and Methods

### 4.1. Human DPSCs Isolation and Immune Selection

This study was carried out in compliance with the recommendations of Comitato Etico Provinciale—Azienda Ospedaliero-Universitaria di Modena (Modena, Italy), which provided the approval of the protocol (ref. number 3299/CE; 5 September 2017). Human DPSCs were isolated from third molars of adult subjects (*n* = 3; 30–35 years) undergoing routine dental extraction. All subjects gave written informed consent in accordance with the Declaration of Helsinki.

Cells were isolated from dental pulp as previously described [23]. Briefly, dental pulp was harvested from the teeth and underwent enzymatic digestion by using a digestive solution, (3 mg/mL type I collagenase plus 4 mg/mL dispase in α-MEM). Pulp was then filtered onto 100 μm Falcon Cell Strainers, in order to obtain a cell suspension. Cell suspension was then plated in 25 cm^2^ culture flasks and expanded in standard culture medium [α-MEM supplemented with 10% heat inactivated foetal bovine serum (FBS), 2 mM L-glutamine, 100 U/mL penicillin, 100 μg/mL streptomycin] at 37 °C and 5% CO2. Following cell expansion, human DPSCs were immune-selected by using MACS^®^ separation kit according to manufacturers’ instructions. The immune-selections were performed by using primary antibodies: mouse IgM anti-STRO-1 and rabbit IgG anti-c-Kit (Santa Cruz, Dallas, TX, USA). The following magnetically labelled secondary antibodies were used: anti-mouse IgM and anti-rabbit IgG (Miltenyi Biotec, Bergisch Gladbach, Germany). The immune-selection resulted in the isolation of a homogeneous hDPSCs population expressing STRO-1 and c-Kit. All the experiments were performed using STRO-1^+^/c-Kit^+^ hDPSCs.

### 4.2. Titanium Surfaces Characterization

A total of 50 titanium disks (MegaGen Co. Ltd., Daegu, South Korea) measuring 13 mm in diameter and 3 mm in thickness were used in this study. Particularly, two different titanium surfaces were used: machined (MCH) and Ca2^+^ incorporated (NCA). The treatment processes are hold by the manufacturer. For this study, titanium surfaces were divided into 3 different experimental groups: (1) control surfaces (Ctrl), (2) surfaces cleaned with Ni-Ti brushes (Brush) and (3) surfaces cleaned by air-polishing with NaHCO_3_ 40 µm (Bic40).

The cleansing of the disks was carried out using two mechanical methods: (1) Nickel-Titanium Brushes (I.C.T. Brush micro, Hans Korea Co. Ltd., Gyeonggi-do, Korea/ De Ore, Verona, Italy) and (2) Air-Polishing System (Combi-Touch, Mectron spa, Carasco, Genova, Italy) with 40 µm bicarbonate powder.

Briefly, the “I.C.T.” (Implant Cleaning Technique) nickel-titanium brushes, made up of about 40 super elastic filaments with a diameter of 0.07–0.13 mm, were used at 400 rpm and 600 rpm, respectively, for two sequential rounds of 45 s each. The total duration of each surface treatment was 90 s and a 25 g pressure calibrated on an electronic scale was used, with 100 N of torque. All the treatments were performed by the same operator under irrigation with buffer saline solution (0.9% NaCl).

The “Combi-Touch” air polishing system with sodium bicarbonate particles (ø 40 µm) was used for 30 s at a distance of 5 mm. In particular, the operating principle of “Combi Touch” air-polishing system consists in the mechanical action of compressed air spreading an accelerated flow of particles onto the titanium surface. When the particles hit the surface, their kinetic energy is dissipated almost completely, thus producing a gentle although effectively cleansing action. The cleaning treatment is completed by a water jet that is arranged in the form of a bell around the main flow and that uses the pressure drop originated around the nozzle to prevent the powder cloud from bouncing and being dispelled and, at the same time, to dissolve the powder by washing the surface.

After the cleansing treatments, surface morphology for each experimental group was qualitatively evaluated through Scanning Electron Microscopy (EVO MA 10—Carl Zeiss, Oberkochen, Germany) working at 25 keV. Moreover, surface roughness was determined by Atomic Force Microscopy (Nanoscope IIIa, Veeco, Santa Barbara, CA, USA) and Ra, Rpv and Rms parameters were obtained. Ra (Roughness average) measures the average surface roughness considering the peaks and the valley means. The Rpv (peak to valley distance) describes the maximum observed range in a sample area and it is given by the distance between the highest peak and the lowest valley on a measured surface. Rms parameter describes the density of micropores on the surface.

### 4.3. Cell Morphology and Proliferation

Undifferentiated STRO-1^+^/c-Kit^+^ hDPSCs at passage 1 were seeded at a density of 2.5 × 10^3^ cell/cm^2^ on titanium disks in 12-multiwell units and expanded under standard culture conditions. After 7 days of culture, cells were fixed in ice-cold paraformaldehyde 4% for 15 min without dissociating them from the titanium disks. The cells were subsequently permeabilized with 0.1% Triton X-100 in PBS for 5 min, stained with AlexaFluor546 Phalloidin (Thermo Fisher Scientific) and rinsed with PBS 1%. Nuclei were stained with 1 µg/mL 4′,6-diamidino-2-phenylindole (DAPI) in PBS 1%. Titanium disks were mounted with DABCO anti-fading medium on cover glasses. Cell proliferation and morphology were assessed using confocal microscopy (Nikon A1), as formerly described by Bianchi et al. [24].

Cell proliferation was measured by counting the DAPI-positive nuclei on 10 randomly selected fields measuring 2.85 × 10^5^ µm^2^ for each disk by a blind operator. At the same time, average cell area was measured on hDPSCs from 10 randomly selected fields, measuring 2.85 × 10^5^ µm^2^, on 3 disks for each experimental group.

### 4.4. Evaluation of Stemness Markers in hDPSCs Cultured on Titanium Disks

After 1 week of culture on each disk, cells were fixed in 4% ice-cold paraformaldehyde in PBS for 15 min and then processed as previously described [31]. The following primary Abs diluted 1:100 were used: mouse IgM anti-STRO-1 and rabbit IgG anti-c-Kit (Santa Cruz, Dallas, TX, USA). Secondary Abs (goat anti-mouse IgM Alexa488, goat anti-rabbit Alexa546) were diluted 1:200 (Thermo Fisher Scientific, Waltham, MA, USA). Nuclei were stained with 1 µg/mL 4′,6-diamidino-2-phenylindole (DAPI) in PBS 1%. The multi-labelling immunofluorescence experiments were carried out avoiding cross-reactions between primary and secondary Abs.

Confocal imaging was performed using a Nikon A1 confocal laser scanning microscope, as previously described [32]. The confocal serial sections were processed with ImageJ software to obtain 3-dimensional projections and image rendering was performed by Adobe Photoshop Software.

### 4.5. Microbial Strain

We used the bioluminescent *Pseudomonas aeruginosa* strain (P1242) (BLI-*Pseudomonas*) previously engineered in order to express the luciferase gene and substrate under the control of a constitutive P1 integron promoter 2 [33]; thus, live cells constitutively produce a detectable bioluminescent signal. To quantify the bioluminescence emission by BLI-Pseudomonas in the experimental groups, a calibration curve was created allowing to express such values in terms of colony forming units (CFU)/mL; in particular, serial dilutions (starting from 1 × 10^8^/mL) of a bacterial suspension in Tryptic Soy Broth (TSB) (OXOID, Milan, Italy) with 2% sucrose were prepared and a volume of 100 μL of each dilution was seeded in a black-well microtiter plate. The plate was immediately read by using a Fluoroskan Luminescence reader (Thermo Fisher Scientific, Waltham, MA, USA).

### 4.6. Biofilm Formation onto Titanium Disks

In order to allow biofilm formation onto Ca-structured titanium disks, 180 µL of overnight cultures of BLI-Pseudomonas (10^6^/mL) in TSB plus 2% sucrose were seeded in 96 black well-plate, containing 1 disc/well. In parallel, BLI-Pseudomonas was seeded in wells without the titanium disks. The plates were then incubated at 35 °C for 24 h, into the Fluoroskan reader and the bioluminescence was detected at every hour to evaluate in real time, the total microbial load. After incubation, the disks were washed twice with phosphate buffered saline (PBS) (EuroClone, Wetherby, UK) at room temperature (RT), transferred into new wells and the bioluminescence signal was again measured; the obtained values were referred to the amounts of biofilm formed onto disk surfaces. Through the calibration curve, the relative luminescent units (RLU) obtained in each experiment were converted in CFU/mL.

### 4.7. Biofilm Re-Growth onto Treated and Untreated Titanium Disks

Following biofilm formation, the disks were split in two groups and the cleaning treatment was performed, as detailed above; then, controls (untreated) and cleaned (exposed to Bic40 µm for 30 s/surface) were transferred into new wells containing fresh medium and further assessed for microbial residual load and time-related re-growth. Briefly, treated and untreated disks were analysed by Fluoroskan reader, immediately (residual biofilm) and at any hour during a further 24 h incubation at 35 °C. Through the calibration curve, the RLU were converted in CFU/mL.

### 4.8. Statistical Analysis

All experiments were performed in triplicate. Data were expressed as mean ± standard deviation (SD). Differences between two experimental conditions were analysed by paired, Student’s *t*-test. Differences among three or more experimental samples were analysed by ANOVA followed by Newman–Keuls post hoc test (GraphPad Prism Software version 5 Inc., San Diego, CA, USA). In any case, *p* value < 0.05 was considered statistically significant.
